# Artificial intelligence and clinical informatics in UK ophthalmology training: a national cross-sectional survey

**DOI:** 10.1186/s12909-026-09391-5

**Published:** 2026-05-21

**Authors:** Thomas Nash, Rishi Ramessur, Alan Roy Abraham, Peter Thomas

**Affiliations:** 1 Moorfields Eye Hospital NHS Foundation Trust, London, UK; 2 School of Informatics, University of Edinburgh, Edinburgh, EH8 9LE UK; 3 Bristol Eye Hospital, Bristol, UK; 4 University Hospitals Bristol and Weston NHS Foundation Trust, Bristol, UK; 5 NIHR Biomedical Research Centre for Ophthalmology, UCL Institute of Ophthalmology, London, UK

**Keywords:** Artificial intelligence, Clinical informatics, Ophthalmology Medical education, Postgraduate training, Digital health

## Abstract

**Background:**

Artificial intelligence (AI) and clinical informatics (CI) are strategic priorities for UK ophthalmology, with the Royal College of Ophthalmologists identifying education and training as central to safe implementation. However, formal CI/AI training is not currently embedded within the Ophthalmic Specialty Training (OST) curriculum. This study aimed to evaluate trainees’ baseline knowledge, access to training and perceived educational needs to inform future curriculum development.

**Methods:**

We conducted a national cross-sectional online survey of UK ophthalmology trainees between January and June 2025. A bespoke questionnaire was developed in accordance with the CHERRIES framework and informed by national policy and the NHS England DART-Ed digital competency framework. Content validity was strengthened through expert review by members of the Royal College of Ophthalmologists Informatics Working Group. The anonymised survey assessed baseline knowledge, perceived clinical relevance, access to training, educational preferences and career intentions. Quantitative data were analysed descriptively and free-text responses underwent inductive thematic analysis.

**Results:**

Seventy-one trainees responded, representing all UK training deaneries and all training grades. Most reported only basic or no knowledge of CI (76%) or AI (76%), with limited access to deanery-level training. Support for formal education was high (CI 70%; AI 80%). Nearly half (49%) wished to incorporate CI/AI into future consultant roles. Trainees favoured blended, clinically relevant training models combining online learning, regional teaching and supervised project work. Qualitative themes emphasised foundational literacy, governance and safety, practical application and flexible delivery.

**Conclusions:**

UK ophthalmology trainees demonstrate majority support for structured CI/AI education, with over 70% supporting formal training in both domains, despite limited current provision. These findings support integration of digital competencies within the OST curriculum, aligned with national frameworks, to prepare the future workforce for safe and effective AI-enabled ophthalmic care.

**Supplementary Information:**

The online version contains supplementary material available at 10.1186/s12909-026-09391-5.

## Introduction

Ophthalmology is one of the highest-volume NHS specialties, delivering over 7.5 million outpatient appointments and 500,000 surgical procedures annually [1]. Rising demand, driven by an ageing population and increasing prevalence of chronic eye disease continues to place sustained pressure on services. The Royal College of Ophthalmologists (RCOphth) projects a 44–68% rise in the prevalence of common age-related eye conditions by 2035 [1]. This trajectory, further exacerbated by service backlogs generated during the COVID-19 pandemic, highlights the need for innovative and sustainable models of care.

Digital transformation has been identified as a central component of the NHS response. The NHS Long Term Plan emphasises a shift from “analogue to digital” care, highlighting the potential of data-enabled technologies to improve diagnostic accuracy, streamline clinical pathways, and optimise workforce capacity [2]. Within ophthalmology, national programmes promote imaging-led pathways, virtual clinics and diagnostic hubs to improve access and efficiency [3].

Artificial Intelligence (AI) represents a particularly significant opportunity given Ophthalmology’s reliance on high-volume imaging and quantifiable disease markers. As automated diabetic retinopathy screening, glaucoma risk prediction models and virtual imaging-led clinics become increasingly embedded within NHS ophthalmic pathways, trainee exposure to digitally mediated workflows is expanding. The RCOphth Position Statement on AI (2024) articulates an ambition for ophthalmology to be at the forefront of safe and effective AI adoption and identifies education and training as central enablers [4].

In parallel, national frameworks including the Topol Review and the NHS England DART-Ed digital competencies framework, informed by horizon-scanning reports on preparing the clinical workforce for AI, emphasise educational pathways to develop foundational digital literacy for all clinicians and provide role-appropriate specialist training to support evaluation, adoption and governance of emerging technologies [5–8].

Despite this momentum, formal Clinical Informatics (CI) and AI education is not currently embedded within the Ophthalmic Specialty Training (OST) curriculum [9]. To address this gap, we conducted the first national survey of UK Ophthalmic Specialty Training (OST) trainees to assess baseline knowledge of CI and AI, perceived clinical relevance, access to existing training opportunities and preferences for future educational provision to support safe adoption and governance. As digital tools become increasingly embedded within routine practice, understanding trainee readiness and priorities is essential to inform curriculum development aligned with national and RCOphth strategic ambitions for safe AI-enabled care.

## Methods

### Study design

We conducted a national, cross-sectional online survey of UK Ophthalmic Specialty Training (OST) trainees between 1 January and 1 June 2025. The survey assessed trainees’ self-rated knowledge of Clinical Informatics (CI) and Artificial Intelligence (AI), perceived clinical relevance, access to training, preferences for future educational provision, and digital career intentions. The study was designed and reported in line with the CHERRIES checklist (Supplementary Material 5).

### Ethics approval and consent to participate

This study was reviewed using the UK Health Research Authority decision tool and classified as a service evaluation. In accordance with UK national guidance, including the UK Policy Framework for Health and Social Care Research, formal approval from an NHS Research Ethics Committee was therefore not required. The project was registered with the Moorfields Eye Hospital NHS Foundation Trust audit/service evaluation department (Department of AI & Clinical Informatics, project number 1621). Participation was voluntary and anonymous. Participants were provided with study information on the survey landing page and completion of the survey was taken to indicate informed consent to participate.

### Survey development and content validity

A bespoke questionnaire was developed in Microsoft Forms, informed by the OST curriculum, the RCOphth Position Statement on AI (2024), the Topol Review, and the DART-Ed digital competency framework. Content validity was strengthened through expert review by members of the RCOphth Informatics Working Group with internal piloting for usability prior to dissemination. Definitions of CI and AI were provided. The final instrument comprised 21 items covering demographics, baseline knowledge, training access, perceived relevance, training preferences, career intentions and optional free text comments (Supplementary Material 1). Survey structure and item branching logic are shown in Supplementary Material 2.

### Recruitment & survey administration

The survey was distributed electronically via email. Training Programme Directors (TPDs) from all 21 UK ophthalmology deaneries were contacted and asked to disseminate the survey via local trainee mailing lists. All OST trainees (ST1–ST7) were eligible. The total number of UK ophthalmology trainees is not fixed and varies throughout the academic year. Based on data from the Royal College of Ophthalmologists’ Training team, approximately 700 trainees are in Ophthalmic Specialty Training at any given time. No incentives were offered. Responses were collected electronically and duplicate submissions were minimised using Microsoft Forms settings (one response per user).

### Data protection

No directly identifying personal data were collected. Access to raw data was restricted to the study team via Microsoft Forms/Excel export permissions.

### Analysis

Data were exported to Microsoft Excel for descriptive analysis. Categorical variables are reported as frequencies and percentages, with Likert responses summarised by distribution across response options. Analyses are based on submitted questionnaires with incomplete submissions excluded a priori. Free-text responses underwent inductive thematic analysis: two authors (TN and RR) independently coded responses, developed preliminary codes and refined themes through discussion, resolving discrepancies by consensus. Item-level response rates for all 21 survey questions are shown in Supplementary Material 3.

## Results

### Participant characteristics

A total of 71 Ophthalmic Specialty Training (OST) trainees responded, representing an estimated response rate of approximately 10% based on a total UK trainee population of 700. All UK ophthalmology deaneries (21/21) were represented, with the largest proportions from London North (*n* = 16) (Supplementary Material 4. Table S1). Respondents spanned all training grades, with a balanced distribution across junior (ST1–3: *n* = 28, 39%)**,** intermediate (ST4–5: *n* = 20, 28%) and senior (ST6–7: *n* = 23, 32%) trainees (Table 1). When responses were explored by training grade, support for AI training was observed across all levels, with the majority of trainees in most groups reporting a perceived role for formal education. While some variation was noted between grades, including a higher proportion of uncertainty among ST3 trainees, no consistent pattern was identified to suggest that interest was confined to a particular stage of training.

**Table 1:** Distribution of respondents by training grade

Training Grade	n	%
ST1	11	15.5
ST2	8	11.3
ST3	9	12.7
ST4	10	14.1
ST5	10	14.1
ST6	14	19.7
ST7	9	12.7

### Baseline knowledge of clinical informatics and artificial intelligence

Self-rated knowledge of both CI and AI was generally low (Table 2), with most respondents reporting basic understanding or no knowledge (CI: 76.0%; AI: 76.1%).

**Table 2:** Self-rated knowledge of CI and AI (*n* = 71)

Knowledge level	Clinical Informatics n (%)	Artificial Intelligence n (%)
No knowledge	15 (21.1)	7 (9.9)
Basic understanding	39 (54.9)	47 (66.2)
Intermediate knowledge	14 (19.7)	9 (12.7)
Advanced knowledge	3 (4.2)	8 (11.3)

### Access to training opportunities

Access to deanery-level training was limited (Table 3). Over half reported no available training in CI (56.3%) or AI (54.9%) and no deanery was reported to offer comprehensive training in either domain.

**Table 3:** Deanery level Training opportunities in CI and AI (*n* = 71)

Training provision	CI n (%)	AI n (%)
None available	40 (56.3)	39 (54.9)
Limited (single session)	24 (33.8)	29 (40.8)
Fair (multiple sessions, single modality)	7 (9.9)	3 (4.2)
Comprehensive	0 (0.0)	0 (0.0)

### Demand for formal training & perceived clinical relevance

**Fig. 1:**
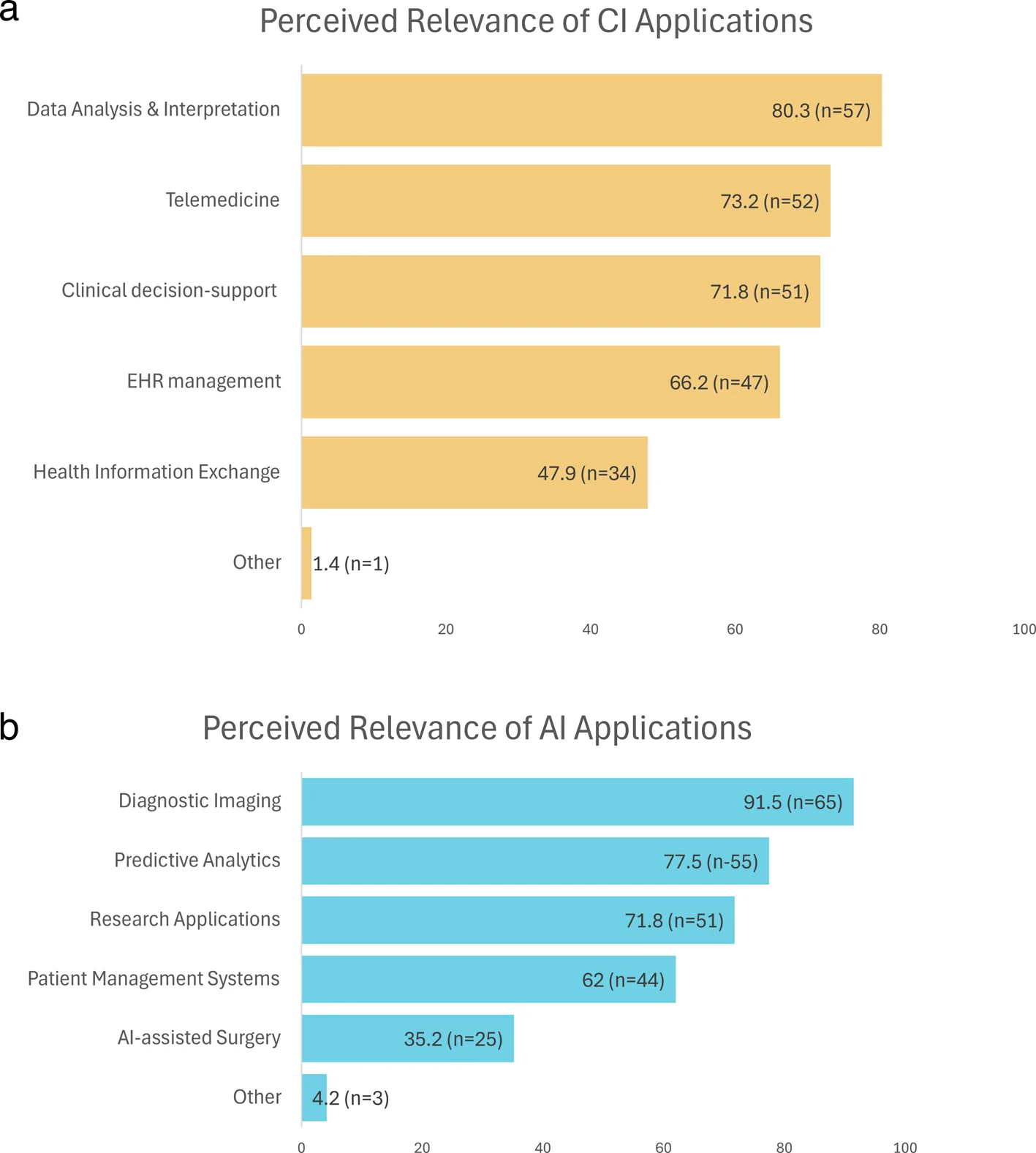
Perceived relevance of (**a**) clinical informatics applications and (**b**) artificial intelligence applications (*n* = 71)

Support for formal training was high (Supplementary Material 4. Table S2): 70.4% supported CI training and 80.3% supporting AI training. Perceived relevance of applications is shown in Fig. 1: for CI, commonly selected areas include data analysis/interpretation (80.3%), telemedicine (73.2%), decision-support (71.8%) and electronic health record (EHR) management (66.2%). For AI, diagnostic imaging was most commonly selected (91.5%), followed by predictive analytics (77.5%), research applications (71.8%) and patient management systems (62.0%).

### Career intentions relating to digital practice

Nearly half (49.3%, 35/71) wished to include CI/AI in their future consultant job plan; 39.4% were unsure (Table 4a). Among those interested, most favoured a subspecialty consultant role with a secondary CI/AI interest supported by a small number of dedicated sessions (71.4%; Table 4b). Interest in more advanced training was mixed (Yes: 36.6%; No 43.7%; Table 4c).

**Table 4:** a Desire to include CI/AI in a future consultant job plan (*n* = 71) (b) Preferred model of future consultant job plan amongst those answering ‘Yes’ (*n* = 35) (c) Interest in fellowship (Level 4) training in CI/AI (*n* = 71)

(a)		
**Response**	**n**	**%**
Yes	35	49.3
Unsure	28	39.4
No	8	11.3

(b)		
**Model**	**n**	**% of “Yes”**
Consultant subspecialty ophthalmologist with a secondary interest in CI/AI and a few dedicated sessions	25	71.4
Consultant ophthalmologist with primary specialist interest in CI/AI and most sessions in these fields	6	17.1
Consultant subspecialty ophthalmologist with an interest in CI/AI but no dedicated sessions	2	5.7
Consultant in CI/AI only	2	5.7
Consultant subspecialty ophthalmologist with a secondary interest in CI/AI and a few dedicated sessions	25	71.4

(c)		
**Response**	**n**	**%**
Yes	26	36.6
Unsure	14	19.7
No	31	43.7

### Preferred educational formats

Two-thirds (66.2%, 47/71) supported embedding CI/AI training within the OST curriculum via subspecialty rotations (Supplementary Material 4. Table S3a). Respondents favoured a blended approach to delivery, most commonly regional teaching sessions (60.6%), structured online courses (59.2%), clinical placements/in-programme fellowships (53.5%) and supervised project-based work (45.1%; Supplementary Material 4. Table S3b).

### Qualitative findings

Sixty free-text responses were provided (Supplementary Material 3) and pooled across questions to identify cross-cutting themes. Five overarching themes were identified:

1. Foundational Literacy: baseline CI/AI literacy as a core competency viewed as integral to safe clinical practice.
2. Clinical Relevance: preference for applied training linked to ophthalmic workflows (e.g., imaging pathways, EHRs, triage and decision-support).
3. Governance & Safety: emphasis on limitations, ethics, bias and critical appraisal of AI outputs.
4. Delivery Model: support for flexible, blended and accessible training (online learning, regional teaching and supervised project work).
5. Career Integration: expectation that CI/AI will increasingly form part of consultant roles, commonly as secondary interests supporting leadership, innovation, research and governance.

## Discussion

### Principal findings

This national survey provides the first systematic evaluation of UK ophthalmology trainees’ knowledge, perceptions, and educational needs relating to Clinical Informatics (CI) and Artificial Intelligence (AI). Trainees reported low baseline knowledge and limited access to formal deanery-level training but recognised the clinical relevance of CI/AI to contemporary ophthalmic practice. Support for structured education was high, particularly for blended learning models combining online teaching, supervised project work and clinically embedded experience. Nearly half of trainees wished to incorporate CI/AI in their future consultant roles, typically as a secondary interest, reflecting evolving workforce expectations as digital technologies become increasingly integrated into routine care.

### Digital transformation and the need for workforce capability

Digital transformation is considered central to NHS plans to address rising demand, constrained workforce capacity and increasing care complexity, with AI and data-enabled workflows offering opportunities to support diagnostic accuracy, efficiency and service redesign. Ophthalmology, as a high-volume imaging-dependent specialty, is a particularly relevant setting for digital innovation. However, effective and safe deployment depends not only on technological capability but on a workforce able to evaluate, govern and use digital tools. This study demonstrates that trainees recognise this need but currently lack structured educational provision to support it.

### AI and clinical informatics in ophthalmology: research maturity, implementation barriers & workforce readiness

Ophthalmology is often considered well positioned for AI adoption, supported by extensive research in diabetic retinopathy screening [10], glaucoma progression prediction [11], and retinal biomarker analysis for systemic disease risk [12]. Despite this, NHS deployment remains limited, reflecting practical barriers to implementation such as bias and generalisability, limited transparency/explainability, medico-legal accountability, interoperability, workflow integration and variable organisational digital maturity.

Importantly, AI systems are not infallible. Performance may degrade when applied to populations differing from training data and may drift over time. Many tools lack transparency or explainability (‘black boxes’), limiting clinicians’ ability to appraise outputs. Additional concerns relate to cost-effectiveness and the wider ethical, social and environmental implications of large-scale digital deployment. Several review articles have described these concerns [13–15].

Rather than diminishing the potential value of AI, these limitations reinforce the need for appropriately trained clinicians. AI tools should function as adjuncts to, rather than replacements for, clinical judgement, with clinicians remaining accountable for decisions informed by AI outputs. Education in AI principles, limitations, evaluation, ethics and governance is therefore required to mitigate risks such as automation bias and inappropriate deployment – which risk patient safety – and are consistent with recommendations by the Alan Turing Institute and General Medical Council [16].

In parallel, CI capability is essential to delivering digital transformation in ophthalmology, including optimisation of electronic health records, interoperable data flows (including imaging) and implementation of virtual and remote care pathways. Infrastructure limitations and interoperability challenges can directly affect efficiency, data quality and patient safety [17–20].

Together, these findings support the need for structured training that spans both AI competencies (critical appraisal, governance and safe use of decision-support tools) and core CI skills (data literacy, workflow optimisation and information governance). The prominence of governance, safety and ethical considerations within the qualitative findings of this study aligns closely with these requirements.

### Alignment with national policy and strategy

Findings reflect national priorities for a digitally capable clinical workforce. The Topol Review and subsequent NHS workforce strategies emphasise the need for progressive digital competency development, while the NHS England DART-Ed framework provides a structured model spanning foundational literacy, governance and advanced capability.

Within ophthalmology, the Royal College of Ophthalmologists’ Position Statement on AI articulates a clear ambition for the specialty to lead in safe and effective AI adoption, identifying education and training as central enablers. This study provides empirical evidence of trainee demand for structured postgraduate CI/AI training to translate strategic ambition into practical workforce capability.

### Comparison with other specialties and the wider education landscape

Training gaps in CI/AI are not unique to Ophthalmology. Surveys across undergraduate and postgraduate medical education consistently demonstrate low baseline digital literacy alongside significant support for formal training [21–25]. In radiology, similar concerns about inadequate AI preparation have informed curriculum updates and the introduction of blended learning programmes [26, 27]. Comparable patterns have emerged in general practice, where use of generative AI tools is increasing despite the absence of formal structured curricular training [28, 29]. Gaps exist in other specialties with high potential for meaningful AI deployment including dermatology and emergency medicine.

Collectively, these findings suggest that digital skills deficits are systemic rather than specialty specific. However, given ophthalmology’s reliance on imaging and early engagement with automated diagnostic pathways, the need for structured CI/AI education may be particularly pressing.

### Interpretation of trainee priorities and implications for curriculum development

Trainees identified broad relevance of CI and AI across imaging, decision-support, workflow optimisation and telemedicine and many viewed digital competencies as integral to future consultant practice. Qualitative analysis highlighted five consistent themes: foundational literacy, preference for clinically relevant applied content, emphasis on governance and safety, support for flexible blended delivery and recognition of digital skills as part of future consultant roles. Equity concerns regarding variable access to training across deaneries further support the need for nationally coordinated approaches.

Curriculum development must also acknowledge opportunity cost within an already demanding training programme as well as heterogeneity of aspirations, with a minority of respondents expressing no interest in AI/CI training and many expressing limited interest in advanced training.

These findings support a tiered model: foundational CI/AI competencies embedded for all trainees – reflecting the universal need for digital literacy and safe use of CI/AI systems—with advanced opportunities for those pursuing specialist or leadership roles. This model aligns with both the RCOphth curriculum structure and the DART-Ed framework. The Medical Training Review Phase 1 diagnostic report similarly emphasises training flexibility and responsiveness to evolving service needs, supporting modular approaches to emerging competency areas such as CI/AI [30].

While trainees represent a key stakeholder group in postgraduate education, curriculum development requires engagement with a broader range of stakeholders including Training Programme Directors, educational supervisors, the Royal College of Ophthalmologists, healthcare organisations and patients. The findings of this study should therefore be interpreted as informing, rather than determining, future curriculum development. Future work should include stakeholder consultation and pilot evaluation of educational interventions to assess feasibility, educational value and impact on clinical practice and patient safety.

### Strengths and limitations

Strengths include national coverage across all UK deaneries, representation across training grades, mixed quantitative and qualitative methods and expert review to support content validity. Limitations include a modest response rate (approximately 10%), potential selection bias, reliance on self-reported knowledge and variable depth of qualitative responses. The response rate is estimated based on national trainee numbers provided by the Royal College of Ophthalmologists, which vary over time and are not fixed at a single timepoint. Moreover, gender was not collected, so differences in perceptions by gender could not be assessed. As an exploratory needs assessment, the study establishes a foundation for further educational research rather than definitive competency measurement.

In summary, this national survey identifies a clear but addressable gap between strategic ambitions for AI-enabled ophthalmic care and current postgraduate training provision. Trainees report limited baseline knowledge and minimal access to structured education yet demonstrate majority support for structured CI/AI education, with over 70% supporting formal training in both domains, alongside strong recognition of clinical relevance and preference for blended, practice-focused training. Embedding foundational CI/AI competencies within the OST curriculum, aligned with national digital frameworks and supported by optional advanced pathways for interested trainees, represents a proportionate and scalable approach to workforce preparation. Future work should evaluate pilot educational interventions and define measurable competency standards to support safe and sustainable implementation.

## Supplementary Information

Supplementary Material 1. Survey questionnaire / survey instrument.

Supplementary Material 2. Survey structure and branching logic.

Supplementary Material 3. Item-level response rates and free-text responses.

Supplementary Material 4. Tables S1-S3a and b.

Supplementary Material 5. CHERRIES checklist

Supplementary Material 6. STROBE statement

## Data Availability

De-identified survey data are available from the corresponding author on reasonable request.

## Abbreviations

AI: Artificial intelligence
CI: Clinical informatics
OST: Ophthalmic Specialty Training
NHS: National Health Service
