# Supplementary material for: Artificial intelligence and clinical informatics in UK ophthalmology training: a national cross-sectional survey
Source: BMC Med Educ. 2026 May 21;26:1343. doi: 10.1186/s12909-026-09391-5 (PMC13491797; doi:10.1186/s12909-026-09391-5)
Supplement: Supplementary file 1 [file 12909_2026_9391_MOESM1_ESM.pdf]

Artificial Intelligence & Clinical Informatics: An OST Training Survey

Introduction
The Royal College of Ophthalmologists recently published a position statement indicating its intention to place Ophthalmology 'at the forefront of AI in healthcare'[1]. It presents 'Education and Training' as one of several required steps to help to fully realise this potential. This National Trainee Survey is designed to gauge current levels of understanding of Clinical Informatics and Artificial Intelligence amongst the national trainee cohort, including their relevant applications to Ophthalmology. It also aims to determine levels of interest in further training and education of these topics. Your feedback may help to identify potential areas for training and development as part of the Ophthalmic Specialist Training curriculum.

Definitions
For the purposes of this survey, please see the below definitions of Artificial Intelligence and Clinical Informatics:

- **Artificial Intelligence (AI)** is a branch of computer science involved in the development of systems that seek to simulate human intelligence. These computer systems can perform tasks such as learning, decision-making, and language understanding. In healthcare, AI has many applications. Such applications include screening for and diagnosing diseases, predicting patient outcomes, personalising treatment plans, automating administrative tasks and optimising operational service efficiency, improving the efficiency of clinical research and enhancing surgical training.

- **Clinical Informatics** is the application of information technology and data management in the healthcare setting to improve patient care. It involves the use of electronic health records (EHRs), data analysis, telemedicine, and decision support systems to enhance the efficiency, accuracy, and quality of clinical practice. Clinical Informatics helps in managing patient information, facilitating communication among healthcare providers, and supporting clinical decision-making processes.

The survey is anonymous and will take approximately **5 minutes to complete**. Thank you for your participation. Your feedback is invaluable in helping us design a curriculum that meets your needs and prepares you for the future of

Section 1: Personal Information

1. What is your current training grade? *

- ◯ ST1
- ◯ ST2
- ◯ ST3
- ◯ ST4
- ◯ ST5
- ◯ ST6
- ◯ ST7

2. In which Deanery do you currently train? *

- East Midlands
- East of England
- East of Scotland
- Kent, Surrey, Sussex (KSS)
- London North
- London South
- Mersey
- North East
- North Scotland
- North West
- Northern Ireland
- Oxford
- Severn
- South West Peninsula
- South-East Scotland
- Wales
- Wessex
- West Midlands
- West Scotland
- Yorkshire & Humberside, North
- Yorkshire & Humberside, South

Section 2: Baseline Knowledge & Understanding

3. How would you rate your current knowledge of: *

	No knowledge	Basic Understanding	Intermediate Knowledge	Advanced Knowledge
Clinical Informatics	○	○	○	○
Artificial Intelligence	○	○	○	○

Section 3: Training Needs

4. How would you rate your current level of training or opportunity to engage with Clinical Informatics in your deanery? *

- ◯ None Available
- ◯ Limited e.g., a single training/teaching session
- ◯ Fair e.g., multiple training/teaching sessions employing only one training delivery method (e.g. regional teaching sessions) on a single topic
- ◯ Comprehensive e.g., multiple training/teaching sessions employing multiple training delivery methods (e.g., regional teaching sessions, online courses, supervised project-based work etc) covering a range of topics

5. How would you rate your current level of training or opportunity to engage with Artificial Intelligence in your deanery? *

- ◯ None Available
- ◯ Limited e.g., a single training/teaching session
- ◯ Fair e.g., multiple training/teaching sessions employing only one training delivery method (e.g. regional teaching sessions) on a single topic
- ◯ Comprehensive e.g., multiple training/teaching sessions employing multiple training delivery methods (e.g., regional teaching sessions, online courses, supervised project-based work etc) covering a range of topics

6. Do you feel there is a role for specific training in Clinical Informatics? *

- ◯ Yes
- ◯ No
- ◯ Unsure

7. Free text Box: Please provide any additional comments explaining your selection.

8. Do you feel there is a role for specific training in Artificial Intelligence? *

- ◯ Yes
- ◯ No
- ◯ Unsure

9. Free text Box: Please provide any additional comments explaining your selection.

10. Which areas of Clinical Informatics do you think are most relevant to your training? (Select all that apply) *

- [ ] Electronic Health Records (EHR) management
- [ ] Data Analysis & Interpretation
- [ ] Health Information Exchange
- [ ] Telemedicine
- [ ] Clinical Decision Support Systems
- [ ] Other (please specify)

11. Free Text box: Please provide any additional comments.

12. Which areas of Artificial Intelligence do you think are most relevant to your training? (Select all that apply) *

- [ ] Diagnostic Imaging
- [ ] Predictive Analytics
- [ ] AI-assisted surgery
- [ ] Patient Management Systems
- [ ] Research Applications
- [ ] Other (please specify)

13. Free text box: Please provide any additional comments

Section 4: Career Aspirations

14. Ideally, would you like Clinical Informatics or AI to be part of your eventual Consultant job plan? *

- ◯ Yes
- ◯ No
- ◯ Unsure

15. If 'Yes', please select which of the following options would be favourable:

- ◯ Consultant in Clinical Informatics or AI, focusing exclusively on these fields in all sessions.
- ◯ Consultant Ophthalmologist with primary specialist interest in Clinical Informatics or AI focusing exclusively on these fields in most sessions.
- ◯ Consultant Subspecialty Ophthalmologist with a secondary interest in Clinical Informatics or AI focusing exclusively on these fields in a few sessions
- ◯ Consultant Subspecialty Ophthalmologist with an interest in Clinical Informatics and/or AI but without any dedicated sessions

16. Would you consider pursuing a fellowship (Level 4 training) in Clinical Informatics or AI? *

- ◯ Yes
- ◯ No
- ◯ Unsure

Section 5: Preferred Training Methods

17. How would you prefer Clinical Informatics and/or AI training to be delivered? (Select all that apply) *

- ☐ Embedded into the 2024 RCOphth Curriculum through subspecialty training (e.g., project-based work as part of rotations in Community Ophthalmology, Medical Retina, Glaucoma, etc.)
- ☐ As a cross-specialty dual-accredited specialty
- ☐ As an Out of Programme Experience (OOPE)
- ☐ As a post-CCT fellowship
- ☐ I do not believe that training in Clinical Informatics or AI is necessary
- ☐ Other (please specify in the free text box)

18. Free text Box: Please provide any additional comments

19. What methods of training delivery would you prefer for Clinical Informatics and/or AI? (Select all that apply) *

- ☐ Degree/Accredited Course
- ☐ Clinical placement/In-programme fellowship
- ☐ Online courses (e.g., via INSPIRE platform)
- ☐ Regional teaching sessions
- ☐ Supervised project-based work
- ☐ Mentorship programmes
- ☐ Reading materials and self-study
- ☐ Other (please specify in the free text box)

20. Free text Box: Please provide any additional comments

Section 6: Additional Comments

21. Please provide any additional comments or suggestions regarding the provision of Clinical Informatics and AI training.

This content is neither created nor endorsed by Microsoft. The data you submit will be sent to the form owner.

Microsoft Forms
