# Supplementary material for: Artificial intelligence and clinical informatics in UK ophthalmology training: a national cross-sectional survey
Source: BMC Med Educ. 2026 May 21;26:1343. doi: 10.1186/s12909-026-09391-5 (PMC13491797; doi:10.1186/s12909-026-09391-5)
Supplement: Supplementary file 2 [file 12909_2026_9391_MOESM2_ESM.pdf]

Supplementary Material 2: Survey Instrument Branching Logic

Section	Q	Item (short title)	Response format	Answer options / content	Display logic (branching)
Intro	–	Participant information / definitions	Info page	Study background + definitions of CI and AI + estimated completion time (~5 min)	N/A
1	1	Current training grade	Single choice	ST1–ST7	N/A
1	2	Current deanery	Single choice	21 UK deaneries	N/A
2	3	Self-rated knowledge	Single choice	CI and AI each rated: No knowledge / Basic / Intermediate / Advanced	N/A
3	4	CI training access in deanery	Single choice	None / Limited / Fair / Comprehensive	N/A
3	5	AI training access in deanery	Single choice	None / Limited / Fair / Comprehensive	N/A
3	6	Role for CI training	Single choice	Yes / No / Unsure	N/A
3	7	Comment re CI training role	Free text	Open comment	N/A
3	8	Role for AI training	Single choice	Yes / No / Unsure	N/A
3	9	Comment re AI training role	Free text	Open comment	N/A
3	10	Most relevant CI applications	Multiple choice	EHR; Data analysis; Health information exchange; Telemedicine; Decision support; Other	N/A
3	11	Comment re CI relevance	Free text	Open comment	N/A
3	12	Most relevant AI applications	Multiple choice	Diagnostic imaging; Predictive analytics; AI-assisted surgery; Patient management; Research; Other	N/A
3	13	Comment re AI relevance	Free text	Open comment	N/A
4	14	Include CI/AI in future consultant job plan?	Single choice	Yes / No / Unsure	N/A
4	15	Preferred consultant role model (if yes)	Single choice	CI/AI only; Consultant with primary CI/AI; Subspecialty	**Displayed only if Q14 = "Yes"**

				consultant with secondary CI/AI; Subspecialty consultant interested but no sessions	
4	16	Consider Level 4 fellowship in CI/AI?	Single choice	Yes / No / Unsure	N/A
5	17	Preferred structural training model(s)	Multiple choice	Embedded in curriculum via subspecialty training; Dual-accredited; OOPE; Post-CCT fellowship; Not necessary; Other	N/A
5	18	Comment re structure	Free text	Open comment	N/A
5	19	Preferred delivery method(s)	Multiple choice	Degree/accredited; Clinical placement; Online; Regional teaching; Project-based; Mentorship; Reading/self-study; Other	N/A
5	20	Comment re delivery	Free text	Open comment	N/A
6	21	Additional comments	Free text	Open comment	N/A
