# Supplementary material for: Artificial intelligence and clinical informatics in UK ophthalmology training: a national cross-sectional survey
Source: BMC Med Educ. 2026 May 21;26:1343. doi: 10.1186/s12909-026-09391-5 (PMC13491797; doi:10.1186/s12909-026-09391-5)
Supplement: Supplementary file 3 [file 12909_2026_9391_MOESM3_ESM.pdf]

Supplementary Material 3: Response Rates for All 21 questions (n=71)

Q No.	Question	Closed Responses (n, %)	Free-text Responses (n, %)
**1**	Training grade	71 (100%)	–
**2**	Deanery	71 (100%)	–
**3**	Knowledge of CI & AI (Likert)	71 (100%)	–
**4**	CI training availability	71 (100%)	–
**5**	AI training availability	71 (100%)	–
**6**	Should CI training be provided?	71 (100%)	
**7**	Free-text: justification for CI training view	–	13 (18.3%)
**8**	Should AI training be provided?	71 (100%)	
**9**	Free-text: justification for AI training view	–	21 (29.6%)
**10**	CI relevance (tick-all-that-apply)	71 (100%)	
**11**	Free-text: CI relevance comments	–	2 (2.8%)
**12**	AI relevance (tick-all-that-apply)	71 (100%)	
**13**	Free-text: AI relevance comments	–	4 (5.6%)
**14**	Should CI/AI be in consultant job plan?	71 (100%)	–
**15**	If yes: preferred model	35 (49.3%)*	–
**16**	Fellowship (Level 4) interest	71 (100%)	–
**17**	Preferred structural delivery formats	71 (100%)	
**18**	Free-text: comments on structural formats	–	10 (14.1%)
**19**	Preferred training methods (tick-all-that-apply)	71 (100%)	
**20**	Free-text: comments on training methods	–	4 (5.6%)
**21**	Final comments	–	6 (8.5%)

*Q15 is conditional on answering “Yes” in Q14 → only 35 respondents saw this item.
