# Supplementary material for: Artificial intelligence and clinical informatics in UK ophthalmology training: a national cross-sectional survey
Source: BMC Med Educ. 2026 May 21;26:1343. doi: 10.1186/s12909-026-09391-5 (PMC13491797; doi:10.1186/s12909-026-09391-5)
Supplement: Supplementary file 4 [file 12909_2026_9391_MOESM4_ESM.pdf]

**Deanery**	**n**	**%**
London North	16	22.5
North East	7	9.9
South West Peninsula	5	7.0
Kent, Surrey, Sussex (KSS)	4	5.6
London South	4	5.6
Mersey	4	5.6
Northern Ireland	3	4.2
Wales	3	4.2
North West	3	4.2
East of England	3	4.2
Oxford	3	4.2
Yorkshire & Humberside, South	2	2.8
South-East Scotland	2	2.8
North Scotland	2	2.8
West Scotland	2	2.8
Yorkshire & Humberside, North	2	2.8
East Midlands	2	2.8
West Midlands	1	1.4
Wessex	1	1.4
East of Scotland	1	1.4
Severn	1	1.4

Supplementary Table 1. Distribution of respondents by UK Training Deanery

Domain	Yes n(%)	Unsure n (%)	No n (%)
Clinical Informatics (CI)	50 (70.4)	15 (21.1)	6 (8.5)
Artificial Intelligence (AI)	57 (80.3)	8 (11.3)	6 (8.5)

Supplementary Table 2. Trainee views on the need for formal training in CI and AI (n=71)

Option	n	%
Embedded into OST curriculum via subspecialty rotations*	47	66.2
Out of Programme Experience (OOPE)	23	32.4
Post-CCT fellowship	20	28.2
Cross-specialty dual-accredited specialty	20	28.2
Training not necessary	7	9.9
Other	3	4.2

(a) Structural Training Models

Method	n	%
Regional teaching sessions	43	60.6
Online courses (e.g. via INSPIRE)	42	59.2
Clinical placement / in-programme fellowship	38	53.5
Supervised project-based work	32	45.1
Degree / accredited course	32	45.1
Mentorship programmes	31	43.7
Reading materials and self-study	21	29.6
Other	1	1.4

(b) Preferred Training Delivery Method

Supplementary Table 3. Preferred Structural Training Models (a) and Delivery Methods (b) (n=71)
