# Supplementary material for: Artificial intelligence and clinical informatics in UK ophthalmology training: a national cross-sectional survey
Source: BMC Med Educ. 2026 May 21;26:1343. doi: 10.1186/s12909-026-09391-5 (PMC13491797; doi:10.1186/s12909-026-09391-5)
Supplement: Supplementary file 5 [file 12909_2026_9391_MOESM5_ESM.docx]

**Artificial Intelligence and Clinical Informatics in Ophthalmology: A National Training Survey (CHERRIES CHECKLIST)**

Items not reported in the main manuscript are provided in this CHERRIES checklist in accordance with journal word limits.

**Checklist for Reporting Results of Internet E-Surveys (CHERRIES)**

| ***Checklist Item*** | ***Explanation*** | ***Page Number*** |
| --- | --- | --- |
| Describe survey design | National cross-sectional online survey of UK Ophthalmic Specialty Training (OST) trainees (ST1–ST7) to assess knowledge, perceptions, training access and educational needs relating to Clinical Informatics (CI) and Artificial Intelligence (AI), including quantitative items and optional free-text responses. | 2 |
| IRB approval | Not required. Classified as **service evaluation** using the UK Health Research Authority decision tool. Registered with the Moorfields Eye Hospital NHS Foundation Trust audit/service evaluation team. | 2 |
| Informed consent | Survey landing page included participant information (purpose, voluntary nature, anonymity, approximate completion time). **Completion/submission implied consent.** | 2 |
| Data protection | No directly identifying personal data collected. Data stored within Microsoft Forms and exported to Excel; access restricted to the study team. | 2 |
| Development and testing | A bespoke questionnaire was developed in Microsoft Forms, informed by the OST curriculum, RCOphth Position Statement on AI (2024), Topol Review, and NHS England DART-Ed framework. Content validity strengthened through expert review by the RCOphth Informatics Working Group. Internally piloted by the study team to check usability and technical functionality prior to dissemination. Definitions of CI and AI provided at the outset. | 2 |
| Open survey versus closed survey | **Closed survey.** Link distributed via Training Programme Directors (TPDs) through deanery trainee mailing lists. Link not publicly advertised. | 2 |
| Contact mode | Email distribution via Training Programme Directors across UK deaneries. | 2 |
| Advertising the survey | Not publicly advertised (no open social media recruitment). TPDs provided with a standard dissemination email and survey link. | 2 |
| Web/E-mail | Web-based survey hosted on Microsoft Forms. Participants accessed via emailed link. | 2 |
| Context | Survey completed independently by participants in their own time (not supervised). | 2 |
| Mandatory/voluntary | Voluntary Survey | 2 |
| Incentives | None offered. | 3 |
| Time/Date | Survey open **1 January 2025 to 1 June 2025.** | 2 |
| Randomization of items or questionnaires | None. | Supplementary (CHERRIES) |
| Adaptive questioning | Yes. Conditional display logic used for selected items to reduce respondent burden. | 2 |
| Number of Items | 21 items plus optional free-text fields. | 2 |
| Number of screens (pages) | 21 questions across 6 screens/pages. | Supplementary (CHERRIES) |
| Completeness check | No automated completeness check. Incomplete questionnaires excluded **a priori** from analysis. | 3 |
| Review step | No separate review page prior to submission. | Supplementary (CHERRIES) |
| Unique site visitor | Not available in Microsoft Forms (unique visitor metrics not accessible to investigators). | Supplementary (CHERRIES) |
| View rate (Ratio of unique survey visitors/unique site visitors) | Not calculable (unique site visitor data not available). | N/A |
| Participation rate (Ratio of unique visitors who agreed to participate/unique first survey page visitors) | Not calculable (unique first-page visitor counts not available). | N/A |
| Completion rate (Ratio of users who finished the survey/users who agreed to participate) | Not calculable (denominator metrics not available). Analysis based on **submitted questionnaires**; incomplete submissions excluded. | N/A |
| Cookies used | Not used by investigators. | Supplementary (CHERRIES) |
| IP check | Not performed by investigators. | Supplementary (CHERRIES) |
| Log file analysis | Not performed. | Supplementary (CHERRIES) |
| Registration | Microsoft Forms settings used to minimise duplicate entries **(one response per user).** | 3 |
| Handling of incomplete questionnaires | Incomplete questionnaires excluded **a priori;** analyses based on submitted questionnaires only. | 3 |
| Questionnaires submitted with an atypical timestamp | Not applicable / not assessed. | N/A |
| Statistical correction | Not applicable (descriptive analysis only; no hypothesis testing / multiple comparisons correction required). | N/A |
