# Supplementary material for: Artificial intelligence and clinical informatics in UK ophthalmology training: a national cross-sectional survey
Source: BMC Med Educ. 2026 May 21;26:1343. doi: 10.1186/s12909-026-09391-5 (PMC13491797; doi:10.1186/s12909-026-09391-5)
Supplement: Supplementary file 6 [file 12909_2026_9391_MOESM6_ESM.docx]

STROBE Statement—checklist of items that should be included in reports of observational studies

|  | Item No. | Recommendation | Page  No. | Relevant text from manuscript |
| --- | --- | --- | --- | --- |
| **Title and abstract** | 1 | (*a*) Indicate the study’s design with a commonly used term in the title or the abstract | 1 | ‘cross sectional online survey…developed in accordance with the CHERRIES framework’ |
|  |  | (*b*) Provide in the abstract an informative and balanced summary of what was done and what was found | 1 | ‘We conducted a cross-sectional online survey of UK ophthalmology trainees’ and ‘most reported only basic or no knowledge with limited access to deanery-level training’ |
| Introduction | | | |  |
| Background/rationale | 2 | Explain the scientific background and rationale for the investigation being reported | 2 | ‘Digital transformation has been identified as a central component of the NHS response to these challenges’ and ‘ Despite this policy momentum, formal clinical informatics and AI education has not yet been embedded within the current OST curriculum’ |
| Objectives | 3 | State specific objectives, including any prespecified hypotheses | 2 | ‘This study aimed to assess baseline knowledge of CI and AI, explore perceived clinical relevance, evaluate access to existing training opportunities and identify trainee preferences for future educational provision.’ |
| Methods | | | |  |
| Study design | 4 | Present key elements of study design early in the paper | 2 | ‘We conducted a national, cross-sectional online survey of UK Ophthalmic Specialty Training’ |
| Setting | 5 | Describe the setting, locations, and relevant dates, including periods of recruitment, exposure, follow-up, and data collection | 2 | ‘online survey’…‘between 1 January and 1 June 2025.’…’recruitment& survey administration’… |
| Participants | 6 | (*a*) *Cohort study*—Give the eligibility criteria, and the sources and methods of selection of participants. Describe methods of follow-up  *Case-control study*—Give the eligibility criteria, and the sources and methods of case ascertainment and control selection. Give the rationale for the choice of cases and controls  *Cross-sectional study*—Give the eligibility criteria, and the sources and methods of selection of participants | 2 | ‘All OST trainees (ST1-7)were eligible to participate’ trainees (ST1–ST7) were eligible to participate. No incentives were offered.  ‘The survey was distributed electronically via email. TPDs from each of the 21 UK deaneries were contacted.’ |
|  |  | (*b*) *Cohort study*—For matched studies, give matching criteria and number of exposed and unexposed  *Case-control study*—For matched studies, give matching criteria and the number of controls per case | N/A | Not applicable (cross-sectional survey; no matching). |
| Variables | 7 | Clearly define all outcomes, exposures, predictors, potential confounders, and effect modifiers. Give diagnostic criteria, if applicable | 2 | ‘Informatics Working Group’ ‘demographics, baseline knowledge, training access, perceived relevance, training preferences, career intentions’ |
| Data sources/ measurement | 8* | For each variable of interest, give sources of data and details of methods of assessment (measurement). Describe comparability of assessment methods if there is more than one group | 2 | ‘A bespoke questionnaire was built in Microsoft Forms’  ‘Data protection’ |
| Bias | 9 | Describe any efforts to address potential sources of bias | 2 | ‘Potential sources of bias were mitigated by distributing the survey nationally via multiple trainee networks and deaneries to maximise representativeness across grades and regions. Duplicate responses were minimised using Microsoft Forms settings (one response per user), and the questionnaire underwent expert review to strengthen content validity.’ |
| Study size | 10 | Explain how the study size was arrived at | 2 | Survey was disseminated nationally to all eligible OST trainees; no a priori sample size calculation. |

Continued on next page

| Quantitative variables | 11 | Explain how quantitative variables were handled in the analyses. If applicable, describe which groupings were chosen and why | 3 | Data were exported to Microsoft Excel for descriptive analysis. Categorical variables are reported as frequencies and percentages. Likert responses are summarised by distribution across response options. |
| --- | --- | --- | --- | --- |
| Statistical methods | 12 | (*a*) Describe all statistical methods, including those used to control for confounding | 3 | Data were exported to Microsoft Excel for descriptive analysis. Categorical variables are reported as frequencies and percentages. Likert responses are summarised by distribution across response options. Free-text responses were analysed using inductive thematic analysis |
|  |  | (*b*) Describe any methods used to examine subgroups and interactions | N/A | No subgroup/interaction analyses were performed. |
|  |  | (*c*) Explain how missing data were addressed | 3 | ‘incomplete submissions excluded a priori.’ |
|  |  | (*d*) *Cohort study*—If applicable, explain how loss to follow-up was addressed  *Case-control study*—If applicable, explain how matching of cases and controls was addressed  *Cross-sectional study*—If applicable, describe analytical methods taking account of sampling strategy | 3 | No sampling weights/complex sampling; descriptive analysis of respondents. |
|  |  | (*e*) Describe any sensitivity analyses | N/A | No sensitivity analyses. |
| Results | | | | |
| Participants | 13* | (a) Report numbers of individuals at each stage of study—eg numbers potentially eligible, examined for eligibility, confirmed eligible, included in the study, completing follow-up, and analysed | 3 | incomplete submissions excluded a priori.’  ‘A total of **71** Ophthalmic Specialty Training (OST) trainees responded.’ |
|  |  | (b) Give reasons for non-participation at each stage | N/A | Not captured. As this was an open, voluntary online survey, reasons for non-participation were not collected. |
|  |  | (c) Consider use of a flow diagram | n/a | n/a |
| Descriptive data | 14* | (a) Give characteristics of study participants (eg demographic, clinical, social) and information on exposures and potential confounders | 3 | ‘Participant Characteristics’, ‘Distribution of respondents by training grade’ |
|  |  | (b) Indicate number of participants with missing data for each variable of interest | 3 Supplementary Appendix 3 | incomplete submissions excluded a priori.’ ‘*Q15 is conditional on answering “Yes” in Q14 → only 35 respondents saw this item.’ |
|  |  | (c) *Cohort study*—Summarise follow-up time (eg, average and total amount) | n/a | n/a |
| Outcome data | 15* | *Cohort study*—Report numbers of outcome events or summary measures over time | n/a | n/a |
|  |  | *Case-control study—*Report numbers in each exposure category, or summary measures of exposure | n/a | n/a |
|  |  | *Cross-sectional study—*Report numbers of outcome events or summary measures | 3-4 | Outcome data are reported as summary measures (counts and percentages) for all key survey items, including baseline knowledge of CI/AI, access to training, support for formal education, career intentions and preferred training models (Results; Tables 2–4; Figure 1). |
| Main results | 16 | (*a*) Give unadjusted estimates and, if applicable, confounder-adjusted estimates and their precision (eg, 95% confidence interval). Make clear which confounders were adjusted for and why they were included | N/A | Descriptive results only; no adjusted analyses performed. |
|  |  | (*b*) Report category boundaries when continuous variables were categorized | N/A | Not applicable (no categorisation of continuous variables beyond described categories). |
|  |  | (*c*) If relevant, consider translating estimates of relative risk into absolute risk for a meaningful time period | N/A | N/A |

Continued on next page

| Other analyses | 17 | Report other analyses done—eg analyses of subgroups and interactions, and sensitivity analyses | 4 | All free-text responses were included in analysis and pooled across questions to identify cross-cutting themes. |
| --- | --- | --- | --- | --- |
| Discussion | | | | |
| Key results | 18 | Summarise key results with reference to study objectives | 4 | Principal Findings |
| Limitations | 19 | Discuss limitations of the study, taking into account sources of potential bias or imprecision. Discuss both direction and magnitude of any potential bias | 6 | Strengths and Limitations |
| Interpretation | 20 | Give a cautious overall interpretation of results considering objectives, limitations, multiplicity of analyses, results from similar studies, and other relevant evidence | 6 | Overall, this survey suggests UK ophthalmology trainees perceive CI/AI as increasingly relevant and report high demand for structured education, despite limited current provision and low self-rated knowledge. Findings should be interpreted cautiously given the modest sample size, voluntary participation and potential selection bias, and should be considered alongside wider national digital workforce evidence and policy frameworks (e.g., DART-Ed/Topol) when informing curriculum development. |
| Generalisability | 21 | Discuss the generalisability (external validity) of the study results | 6 | Respondents represented all UK training grades and deaneries, supporting relevance across OST; however, as participation was voluntary, findings may over-represent trainees with greater interest in CI/AI and should be generalised cautiously. |
| Other information | |  | | |
| Funding | 22 | Give the source of funding and the role of the funders for the present study and, if applicable, for the original study on which the present article is based | 7 | ‘This research was funded in whole, or in part, by the UK Research and Innovation (UKRI) Open Access Fund via the University of Edinburgh UKRI AI Centre for Doctoral Training in Biomedical Innovation (EP/Y009460/1). For the purpose of open access, the author has applied a Creative Commons Attribution (CC BY) licence to any Author Accepted Manuscript version arising.’ |

*Give information separately for cases and controls in case-control studies and, if applicable, for exposed and unexposed groups in cohort and cross-sectional studies.

**Note:** An Explanation and Elaboration article discusses each checklist item and gives methodological background and published examples of transparent reporting. The STROBE checklist is best used in conjunction with this article (freely available on the Web sites of PLoS Medicine at http://www.plosmedicine.org/, Annals of Internal Medicine at http://www.annals.org/, and Epidemiology at http://www.epidem.com/). Information on the STROBE Initiative is available at www.strobe-statement.org.
